# Diversification and Molecular Evolution of *ATOH8,* a Gene Encoding a bHLH Transcription Factor

**DOI:** 10.1371/journal.pone.0023005

**Published:** 2011-08-04

**Authors:** Jingchen Chen, Fangping Dai, Ajeesh Balakrishnan-Renuka, Florian Leese, Werner Schempp, Felix Schaller, Michael M. Hoffmann, Gabriela Morosan-Puopolo, Faisal Yusuf, Izak Johannes Bisschoff, Verena Chankiewitz, Jinglun Xue, Jingzhong Chen, Kang Ying, Beate Brand-Saberi

**Affiliations:** 1 Department of Anatomy and Molecular Embryology, Ruhr-University of Bochum, Bochum, Germany; 2 Department of Molecular Embryology, Freiburg University, Freiburg, Germany; 3 Department of Animal Ecology, Evolution and Biodiversity, Ruhr-University of Bochum, Bochum, Germany; 4 Institute of Human Genetics, Freiburg University, Freiburg, Germany; 5 Department of Medicine, Freiburg University, Freiburg, Germany; 6 Institute of Genetics, Fudan University, Shanghai, China; 7 Faculty of Biology, Freiburg University, Freiburg, Germany; Ecole Normale Supérieure de Lyon, France

## Abstract

*ATOH8* is a bHLH domain transcription factor implicated in the development of the nervous system, kidney, pancreas, retina and muscle. In the present study, we collected sequence of *ATOH8* orthologues from 18 vertebrate species and 24 invertebrate species. The reconstruction of *ATOH8* phylogeny and sequence analysis showed that this gene underwent notable divergences during evolution. For those vertebrate species investigated, we analyzed the gene structure and regulatory elements of *ATOH8*. We found that the bHLH domain of vertebrate ATOH8 was highly conserved. Mammals retained some specific amino acids in contrast to the non-mammalian orthologues. Mammals also developed another potential isoform, verified by a human expressed sequence tag (EST). Comparative genomic analyses of the regulatory elements revealed a replacement of the ancestral TATA box by CpG-islands in the eutherian mammals and an evolutionary tendency for TATA box reduction in vertebrates in general. We furthermore identified the region of the effective promoter of human *ATOH8* which could drive the expression of EGFP reporter in the chicken embryo. In the opossum, both the coding region and regulatory elements of *ATOH8* have some special features, such as the unique extended C-terminus encoded by the third exon and absence of both CpG islands and TATA elements in the regulatory region. Our gene mapping data showed that in human, *ATOH8* was hosted in one chromosome which is a fusion product of two orthologous chromosomes in non-human primates. This unique chromosomal environment of human *ATOH8* probably subjects its expression to the regulation at chromosomal level. We deduce that the great interspecific differences found in both *ATOH8* gene sequence and its regulatory elements might be significant for the fine regulation of its spatiotemporal expression and roles of *ATOH8,* thus orchestrating its function in different tissues and organisms.

## Introduction

bHLH transcription factors play very important regulatory roles during embryonic development, e.g. in neurogenesis, myogenesis, hematopoiesis, sex determination, and gut development [1]. In animals, bHLH proteins have been classified into six groups, named A, B, C, D, E, and F, based on their phylogenetic relationships and different biochemical properties [2]. ATOH8 belongs to group A of bHLH transcription factors [1]. Specifically, it is classified as a member of NET family within the atonal superfamily which includes families of NeuroD, Neurogenin, Atonal, Oligo, Beta3, Delilah, Mist and NET [2], [3]. In general, proteins of the atonal family are encoded by one single exon and are involved in neurogenesis [4]. Exceptionally, ATOH8 is encoded by 3 exons and is implicated in multiple developmental events in addition to neurogenesis. In the fruit fly, the *ATOH8* orthologue, *NET,* is involved in the wing vein morphogenesis [5]. In the mouse, *ATOH8 (MATH6)* induces neurogenesis but inhibits gliogenesis in the developing retina [4]. Mouse *ATOH8* is also involved in podocyte differentiation during kidney development [6] and endocrine pancreas development [7]. Inactivation of *ATOH8* results in embryonic lethality in mice [7]. In the zebrafish, *ATOH8* is expressed in the developing retina and somites, and knockdown of *ATOH8* results in malformation of the retina and skeletal muscles [8]. A recent study shows that *ATOH8* inhibits neuronal differentiation in the developing retina in the chicken [9]. Besides, the level of *ATOH8* expression increases in U2OS cells transfected with Cyclin-B1-EGFP fusion gene [10], and altered expression levels of *ATOH8* are detected in human patients who suffer from oligodendrogliomas [11]. An altered *ATOH8* expression level is also reported in glioblastoma multiforme [12]. Considering these multiple implications of *ATOH8* in different biological, developmental and pathological processes, we were intrigued to know if the function and regulation of expression of *ATOH8* are conserved across different evolutionary lineages.

In the present study, we assembled *ATOH8* sequence of different species from the GenBank and our re-sequencing data, analyzed the phylogeny of *ATOH8* and performed multiple sequence comparisons. The results show that among metazoans, ATOH8 has experienced a high sequence divergence which makes inferences on basal metazoan relationships difficult. Within the analyzed vertebrate species, the evolutionary relationship of *ATOH8* gene is mostly compliant with the accepted classification of the analyzed species. The bHLH domain of vertebrate ATOH8 was highly conserved. Mammals developed another potential isoform during evolution. Some amino acids are absent in zebrafish, frog and chicken ATOH8 compared to the mammalian orthologues. Vertebrates had a TATA-box type element that secondarily shifted to CpG-island type in mammals except for the opossum. The opossum ATOH8 displayed an evolved structure with an extended C-terminus, accompanied with an absence of CpG islands and TATA elements in the regulatory region. Gene mapping showed that the *ATOH8* host chromosome, chromosome 2 in human, has two orthologous chromosomes in non-human primates. Experimentally, we identified the effective promoter of human *ATOH8* which could drive the expression of reporter genes in the chicken embryo. To summarize, while ATOH8 maintains the conserved bHLH domain, it shows a high sequence diversification among different evolutionary lineages, in particular when comparing orthologous loci in invertebrate species. This great interspecific diversity may contribute to the functional and regulatory diversification of this gene. Our data demonstrate an example of the diversification pattern of a gene encoding a transcription factor among different species and bring insight into the functional study of *ATOH8* in different animal models.

## Results

### Gene structure of vertebrate *ATOH8*

**Figure 1:**
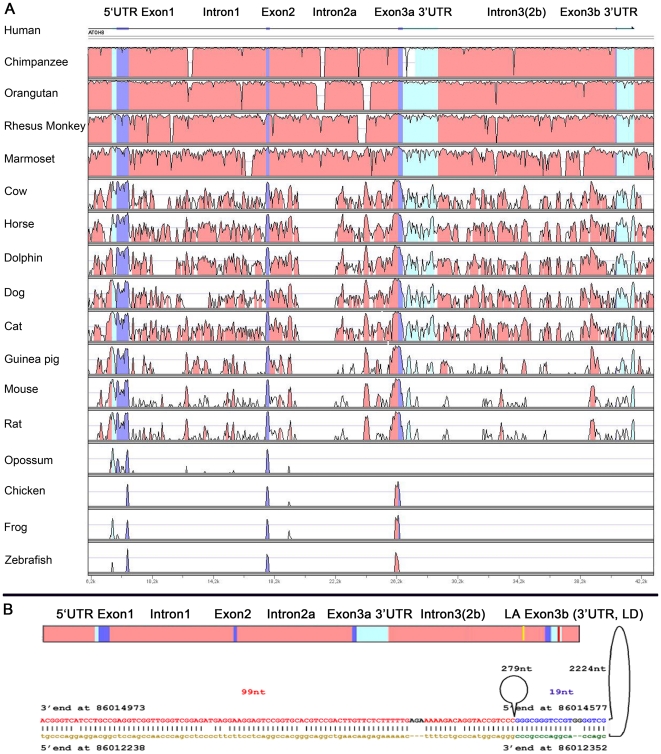
Gene structure of *ATOH8.* (**A**) Interspecies comparisons of *ATOH8* gene loci. In the plot, the horizontal axis represents the sequence of the base genome, and the amplitude represents the degree of sequence conservation. With the *hATOH8* gene locus as a standard reference sequence, the *ATOH8* gene locus is divided into several regions, the exons (blue for protein coding regions and light cyan for UTRs) and the introns (pink). In vertebrates, *ATOH8* consists of three exons (exon1, exon2 and exon3b shown in blue color) and an exon3a is predicted to encode a novel C-terminus of hATOH8 isoform 2. (**B**) A potential DNA loop in primate *ATOH8* gene loci. One fragment is located in the intron between exon3a and exon3b as a potential loop acceptor (LA, marked with yellow); and the second one is located within the exon3b as a loop donor (LD, marked with red). The pink color marks introns, cyan the UTRs and blue the exons. The sketch of potential DNA loop derived from human *ATOH8* is presented in the lower part of the figure.

To determine the gene structure of *ATOH8* in vertebrates, we filled the sequence gaps of *ATOH8* gene loci of seven species (Accession No of Chimpanzee: FN868887.1; cat: FN868891.1; dog: FN868890.1; horse: FN868888.1; dolphin: FN868889.1; rhesus monkey FN868886.1 and chicken: FN868883). With these data supplementing the *ATOH8* genomic sequences from ensemble genome browser (http://www.ensembl.org/index.html), we carried out the genomic sequence analysis among 17 vertebrate species, using the human *ATOH8* genomic sequence as reference (Fig 1A). Our analysis showed that four exons which we termed as exon1, exon2, exon3a and exon3b were present within ATOH8 gene locus of eutherian mammals. *ATOH8* consists of exon1, exon2 and exon3b as annotated in GenBank. Exon3a was verified by an EST (AL831857.1) from the amygdala of the human brain, which with exon1 and exon2 may form another isoform of *ATOH8*. The *ATOH8* gene locus in opossum, chicken, frog and zebrafish contains three exons as well (exon1, exon2 and exon3b), although exon3b shows no significant homology to that of eutherian mammals. We do not know if exon3a exists in these four species or not. In all analyzed species except the opossum, exon3b encodes only one amino acid, the glutamic acid. Instead, exon 3b of the opossum encodes a dozen of amino acids resulting in an extended C-terminus.

With closer analysis we identified a pair of highly complementary sequences in primates, of which one was located in the intron between exon3a and exon3b referred to as loop acceptor (LA), and the complementary sequence was located in exon3b named as loop donor (LD) (Fig 1B and Table S1). This pair of complementary sequences can base-pair each other to potentially introduce a DNA loop (Fig 1B), which might provide additional regulatory facet for *ATOH8* gene in primates.

### Phylogenetic analysis of *ATOH8* gene

**Figure 2:**
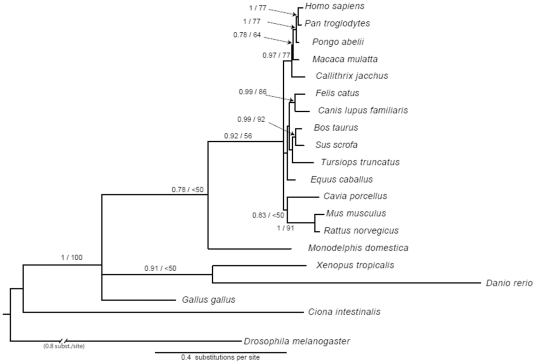
Bayesian phylogenetic tree of *ATOH8* gene. The gene tree was built on the basis of complete coding DNA sequence alignment (2713 bp including gaps) of 20 species. The fruit fly NET gene was used as outgroup. Only branches with a posterior probability of at least 0.75 are shown. Branch support indicates the posterior probabilities and Maximum Likelihood Bootstrap.

To study the evolutionary history of the *ATOH8* gene, we first constructed the phylogenetic tree for the vertebrate *ATOH8* (Fig 2), on the basis of the complete coding sequences of 20 species (Table S2). The translation alignment of the vertebrate *ATOH8* sequences using MAFFT was trivial and resulted in a compact 1,188 bp alignment. When adding the outgroup sequences of the *ATOH8* orthologue, *NET,* from the insect *Drosophila melanogaster* and the sea squirt *Ciona intestinalis,* the alignment was expanded to 2713 bp due to long insertions mainly in *C. intestinalis*. For the nucleotide multiple sequence alignment, jModeltest determined the GTR+G nucleotide substitution model as the most appropriate for the phylogenetic reconstruction.

Bayesian and Maximum Likelihood phylogenetic trees had similar topologies with the Bayesian trees having generally higher support values for the internal nodes. Fig. 2 displays the Bayesian *ATOH8* phylogenetic tree with support values the two different reconstruction methods mapped. For comparison, the Ensembl species tree is also shown (Fig S1 and http://www.phylowidget.org/full/index.html?tree=http://tinyurl.com/ensembltree&useBranchLengths=true&minTextSize=7). The phylogenetic analysis of *ATOH8* resolves a large well-supported vertebrate clade with the major vertebrate taxa only partly resolved as monophyla (e.g. mammals only with posterior probability of 0.78, ML Bootstrap <50, MP Bootstrap 66). Zebrafish as the fish representative is not reported as the most basal vertebrate but rather all major groups (fish, amphibians, mammals, birds) resemble one polyphyletic group in this analysis. Within the mammalian tree, opossum represents the basal group, which is consistent with the species tree. Tip-level phylogenetic relationships are mostly in accordance with the known classification (Primates, Carnivora, Cetartiodactyla, Rodentia) and mostly well-supported by the two methods. The urochordate *Ciona intestinalis* (sea squirt) clusters basal to the vertebrate in group and shows highly-supported affinities to the fruit fly orthologue *NET*.

Extended details regarding the evolution of the *ATOH8* gene was reflected by the Ensembl *ATOH8* gene tree (Fig S2) [13]. Searching in Ensembl genome browser (http://www.ensembl.org/Multi/Search/Results?species=allidx=q=ATOH8), we found the *ATOH8* gene to be present in 36 species which could represent all extant classes of vertebrates. Based on the presently available genomic sequences of *ATOH8* from these species, the *ATOH8* gene tree was generated with the pipeline of gene orthology/paralogy prediction method of Ensembl using only the most conserved regions for analysis (Fig S2). Here, related species are clearly clustered into their respective higher-order taxa in the gene tree, e.g. higher fishes (teleosts) are monophyletic and are resolved as basal vertebrates followed by more derived ‘Amphibia’, ‘Reptilia/Aves’ and ‘Mammalia’; *ATOH8* of chicken and zebrafinch share a common ancestor.

**Figure 3:**
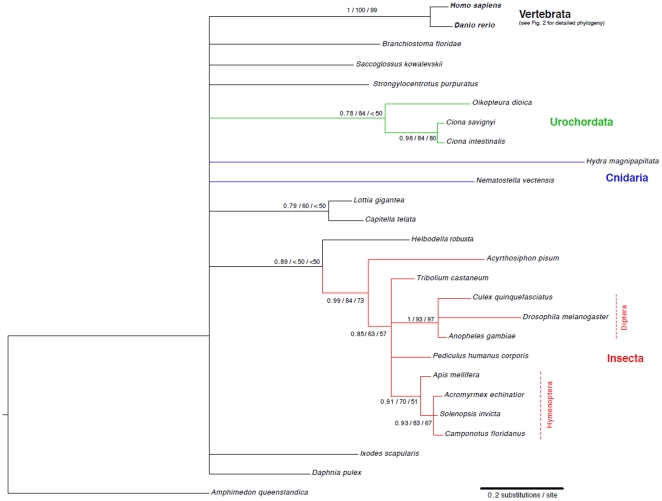
Bayesian phylogenetic tree based on the conserved 82 amino acid alignment of the bHLH domain region. Branches with a posterior probability below 0.75 were collapsed. Support values on the branches resemble the posterior probability values, the PhyML bootstrap, and the RaxML bootstrap values, respectively.

Using *Drosophila melanogaster* NET or *Homo sapiens* ATOH8 as queries, we searched in GenBank and published genome projects to identify possible ATOH8/NET orthologues in invertebrates. Sequences from 22 invertebrate species with a high similarity (Blastp e-values of < = 10^−18^) were collected (Table S3). We did not use sequences from invertebrate species of a genus twice (e.g. just one *Drosophila* species). To reconstruct the phylogenetic relationship of the NET/ATOH8 orthologues among metazoans, we chose the Amq1 sequence (GenBank: ABZ79673.1, Table S3) of the sponge *Amphimedon queenslandica* as outgroup, which resembles the most ancestral protein of Atonal and Twist superfamilies [2]. Human and zebrafish ATOH8 protein sequences were used as vertebrate representatives for this second phylogenetic analysis. The alignments of the 26 different protein sequences using the E-INS-I and the L-INS-I alignment algorithm in MAFFT were different and consisted of many gaps. Both alignments were analyzed using the software gBlocks to search for conserved and conveniently aligned blocks. For both alignments, the same 82 positions were reported from two blocks in the alignments (Table S3) and extracted. The L-INS-I alignment had five additional positions at the N-terminus. However, since no information was available for these positions for some taxa, we used the 82 positions reported from both alignments for subsequent analyses only. Within the 82 bp alignment 20 positions were invariant. Prottest reported the LG+G model as the most appropriate using the BIC and AIC criterion. For the ML calculations using PhyML and RAxML, we used the LG+G model. For Bayesian inferences using MrBayes (which does have the LG model), we used the JTT+G model, which was reported as the best model also implemented in MrBayes. The results of all calculations showed a large basal polytomy from which few well-supported groups branch off (Fig 3). The results of the Bayesian and the two ML analyses were mostly congruent. The vertebrates, urochordates, and insects were correctly resolved as monophyletic groups. Within the insects, the hymenopteran groups ((*Camponotus, Solenopsis, Acromyrmex)* and *Apis*) and the dipterans (*Drosophila, Anopheles, Culex*) were correctly resolved. Also, the holometabolous insects (hymenopterans *+ Tribolium + Pediculus* + dipterans in the tree) were found as the sister group to the hemimetabolous insect representative *Acrythosiphon,* with low support although. In the Bayesian analyses, the two cnidarian species formed a clade (posterior probability of 0.68 only). However, in the ML analyses the two species clustered at two different positions within the tree with very low support although.

### Sequence analysis of the vertebrate ATOH8 protein

**Figure 4:**
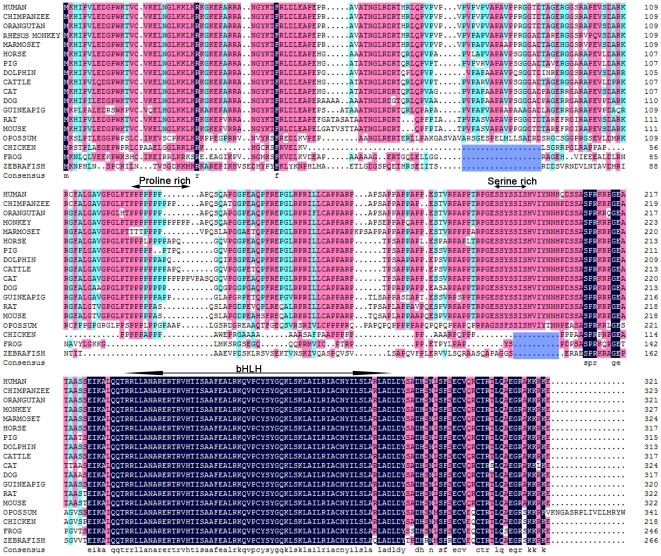
Comparative analysis of 18 vertebrate ATOH8 proteins. The identical amino acids are labeled with black background. Sequences more than 80% conserved are highlighted with carmine. Sequences more than 50% conserved are highlighted with cyan. Dots represent the missing amino acids. The bHLH domain, proline-rich and serine-rich regions are defined with bi-directional arrowheads. The bHLH domain was defined by comparison of the ATOH8 sequence (UniProtKB/Swiss-Prot: Q96SQ7**,** ATOH8_HUMAN) to data from protein database according to the calculation in protein database and protein structure prediction (http://www.compbio.dundee.ac.uk/www-jpred/). The two commonly missing regions in zebrafish, frog and chicken in contrast to mammalian ATOH8 are indicated by blue.

To understand further if *ATOH8* varies among vertebrates, we analyzed the sequence alignments of the ATOH8 protein among the 18 vertebrate species that were collected from GenBank and our re-sequencing data (Table S2). The alignment of protein sequences in vertebrates showed a highly conserved bHLH domain among all species but there were two variable motifs between mammals and non-mammals (Fig 4). The frog and zebrafish lacked the proline-rich motif between the N-terminus and bHLH domain of the protein, and the serine-rich motif was only found in mammals. Among the vertebrates, chicken ATOH8 was the shortest, consisting of 218 amino acids. Dozens of amino acid were absent in the regions approximately 50 amino acid downstream of the N-terminus compared to its vertebrate orthologues. Shorter protein sequence was also observed in zebrafish and frog ATOH8, although not to the same extent as in the chicken. For the opossum, the ATOH8 protein showed a higher similarity to its mammalian orthologues than to the non-mammalian vertebrates. However, it had an extended C-terminus which was encoded by its third exon and was unique among vertebrates.

### *In silico* analysis of regulatory elements of *ATOH8*

**Figure 5:**
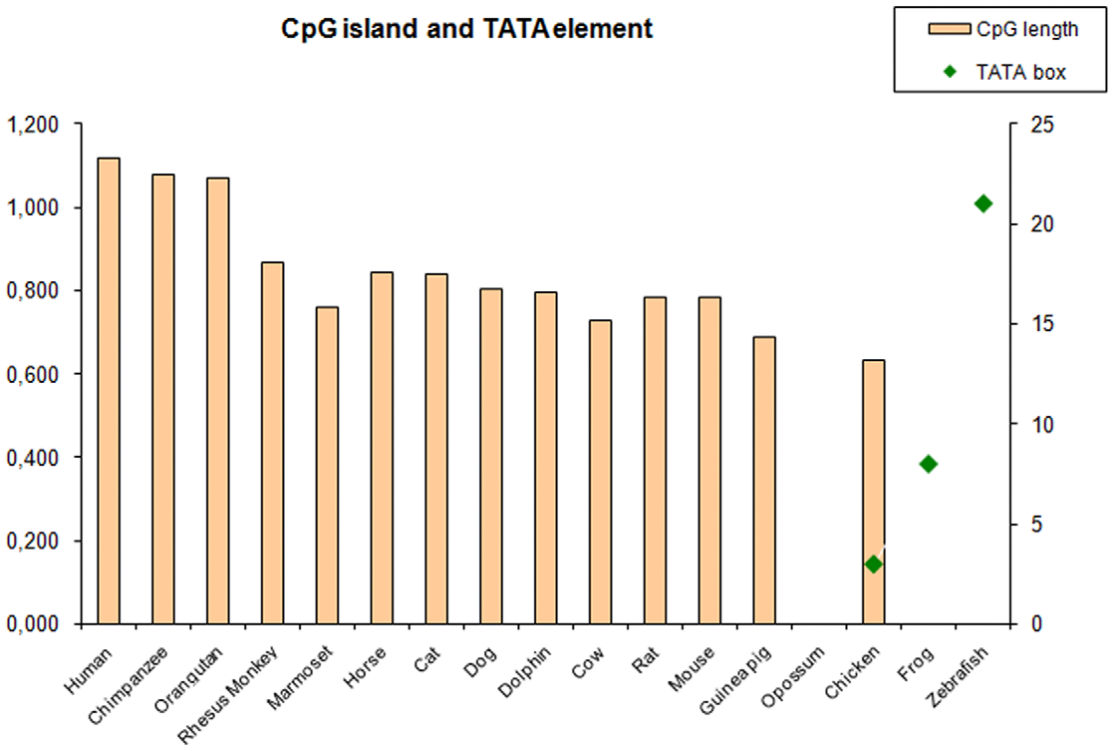
CpG islands and TATA elements in the upstream region of *ATOH8* start codon. In mammals, the upstream region of *ATOH8* coding region is characteristic of CpG islands, of which the human *ATOH8* has the longest CpG islands. No TATA elements (green diamonds) are found. In chicken, both CpG islands and TATA elements are found in *ATOH8* upstream region. In frog and zebrafish, there are no CpG islands but TATA elements present in the upstream region. From chicken, frog to zebrafish, the number of TATA elements increases.

To characterize possible regulatory elements in the upstream region of the *ATOH8* gene, we analyzed regions upstream of the *ATOH8* gene in 17 vertebrate species. The results demonstrated a trend of increased CpG islands size upstream of the *ATOH8* gene in eutherian mammals, where the GC-content in all examined islands was more than 65% (Fig 5). No CpG islands were present in the upstream region of zebrafish and frog *ATOH8*. Instead, we found an abundance of TATA elements in these species (Fig 5, Fig S3). In chicken, both CpG islands and TATA elements were present in the upstream region and the number of TATA elements decreased from zebrafish to frog and chicken (Fig 5). Uniquely, neither CpG islands nor TATA elements were identified in the upstream region of the opossum *ATOH8*.

### Identification of functional regulatory elements of human *ATOH8*

**Figure 6:**
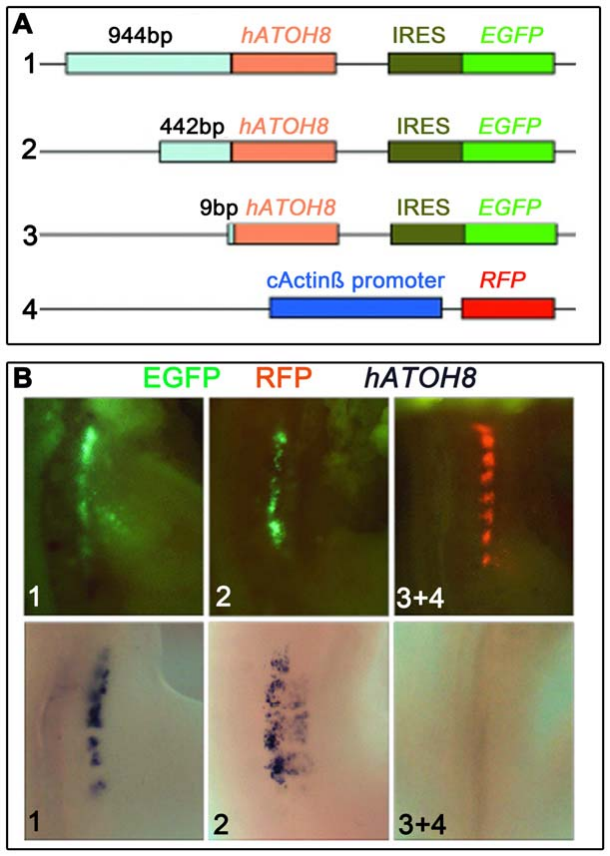
Fragments upstream of human *ATOH8* could drive gene expression in the chicken embryo. (**A**) Diagram of constructed plasmids. 944 nt (1), 442 nt (2) and 9 nt (3) fragments (light cyan) upstream of the *hATOH8* translation start codon, together with *hATOH8* coding sequence (pink) are integrated into pIRES2-EGFP vector by replacement of its CMV promoter, respectively. The cActin-beta-RFP plasmid (4) is used as an additional indicator for effective transfection. (**B**) Exogenous gene expression after transfection of these plasmids into chicken embryos. Both 944 nt (1) and 442 nt (2) fragments could drive the expression of their downstream genes shown by, EGFP (green), and *hATOH8* as shown here after *in situ* hybridization. In contrast, the embryo co-transfected with the control plasmid and cActin-beta-RFP plasmid shows no expression of EGFP or *hATOH8* (3+4).

As *ATOH8* has been found to be deregulated in a number of human diseases [11], [12], we were interested in analyzing the regulatory region of human *ATOH8*. To identify the potential promoter of human *ATOH8* (h*ATOH8*), we isolated a 944 bp and a 442 bp fragment upstream of the *hATOH8* start codon along with the *hATOH8* coding sequence and integrated them into pIRES2-EGFP vector by replacing its CMV promoter (Fig 6A). We transfected these two constructs into the somites of chicken embryos where the chicken *ATOH8* is natively expressed (data not shown), and found that both fragments could drive the expression of EGFP (Fig 6B). To further verify the effect of expression, we analyzed those transfected embryos with a human *ATOH8* probe by *in situ* hybridization and found that human *ATOH8* transcripts were indeed present in the transfected region of the embryos (Fig 6B). In contrast, the control plasmid in which a 46 bp adaptor replaced the CMV promoter, no expression of EGFP or human *ATOH8* transcripts was seen (Fig 6B).

### *ATOH8* gene mapping in primates

**Figure 7:**
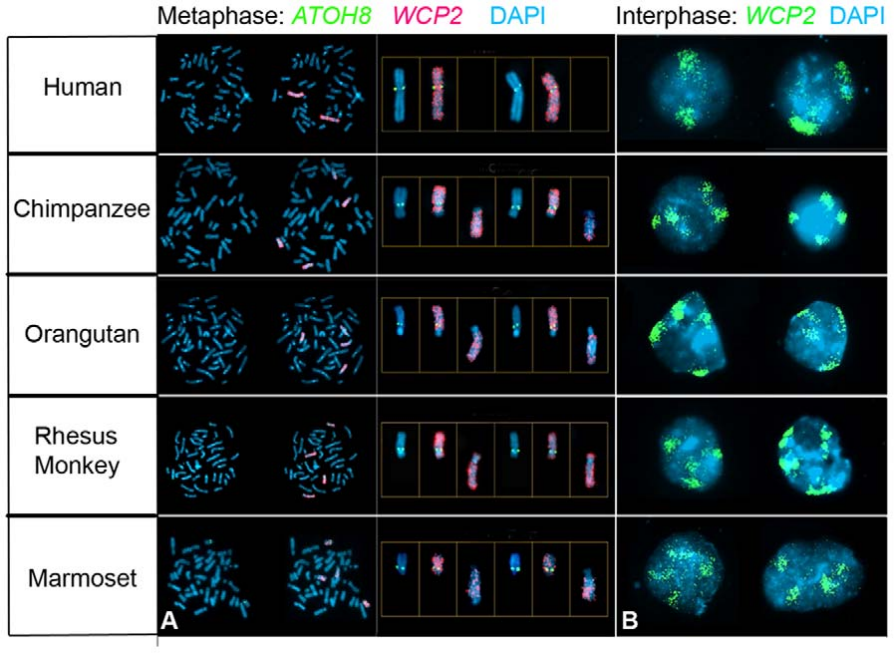
Fish and chromosome painting show the host chromosome of *ATOH8* gene in primates. (**A**) Demonstration of host chromosomes of *ATOH8* in metaphase cells. In human, *ATOH8 (green)* and *WCP2 (red)* staining signals are located on the same two chromosomes. *ATOH8*-positive signal is present on the short arm of *WCP2-*painted chromosomes. In other primates, *ATOH8* signals are positive in two chromosomes, while *WCP2* probe marked four chromosomes, out of which two chromosomes are *ATOH8* positive. (**B**) The territories of *WCP2*-marked chromosomes in interphase cells. In human, *WCP2*-positive chromosomes (green) have two territories in the nucleus, while four separate territories are clearly visible in non-human primates.

The *hATOH8* gene is located in the human chromosome 2, which is a unique human ‘fused’ chromosome. To characterize the orthologues of *hATOH8* host chromosomes and their territories in different primates, we performed fluorescent *in situ* hybridization (FISH) experiments with a human *whole chromosome 2 painting probe* (*WCP2*) and the *hATOH8* gene-specific probe on fixed cells from human, chimpanzee, orangutan, rhesus monkey and marmoset. In metaphase cells, a pair of the human chromosomes was identified from the *WCP2* hybridization signal and the *ATOH8* gene was located in a region of the short arm, which was relatively near the centromere of the painted human chromosomes 2. However, the same probe *WCP2* labelled two pairs of the intermediate-sized ‘non-fused’ chromosomes in non-human primates, and the *hATOH8* signal was only located in the short arm of one pair of the chromosomes which were regarded to be the orthologue of the short arm of human chromosomes 2 (Fig 7a). In the human interphase cell, *WCP2* labeled two chromatin territories, while in the non-human primates four separate territories were visible in the cell nuclei (Fig 7b). The results demonstrate that the short and long arms of human chromosome 2 are restricted to one single chromatin territory, whereas their orthologous chromosomes in non-human primates are separated into two territories.

## Discussion

### The phylogeny of *ATOH8*

The evolution of the vertebrate *ATOH8* gene is mostly in accordance with the known phylogeny of the species analyzed. *NET* of the fruit fly is regarded as an orthologue of vertebrate *ATOH8* [3] and was used together with the *ATOH8* orthologue of *Ciona intestinalis* to root the topology. In the phylogenetic analysis, however, it was not possible to unambiguously align the two orthologues from *Drosophila melanogaster* and *Ciona intestinalis* to the vertebrate ingroup sequences at both nucleotide and protein level. The differences in the alignment had minor effects on branch lengths and support values for the two outgroup taxa relative to another and to the ingroup (results not shown). However, for the vertebrate ingroup which could be aligned consistently, this had no effect. In the phylogenetic trees (Fig. 3) calculated from the 82 amino acid alignment of mainly the bHLH sequences of invertebrates and two vertebrate representatives, the resolution to discriminate between major metazoan lineages (sponges, radialia, protostomes and deuterostomes) is very low. The tree shows many basal polytomies. Even the different chordate lineages do not group together in one monophylum. In other phylogenetic studies based on individual genes, the urochordates were resolved as the sister group to vertebrates, while the cephalochordates represented the most basal chordate lineage [14]. The results of our study hence add no support for either scenario. With this gene it is possible, however, to infer more recent evolutionary events such as the divergence within the vertebrates (Fig. 2) or also within the insects (Fig. 3). Interestingly, the crustacean *Daphnia pulex* NET candidate gene was not resolved basal to the insects, forming a pancrustacean clade, but showed affinities to the sponge sequence. Since about 20% of the genome sequence in *Daphnia pulex* has not been sequenced yet, and prominent gene duplication has been found in this species [15], it is likely, that the *NET* candidate gene found for this species may not be the true *NET* orthologue gene. *Helobdella robusta* clustered basal to the insect group. Due to the very low support value we regard this as an artifactual signal (homoplasy) only. The same seems to be the case for the clade consisting of *Lottia gigantea* and *Capitella telata*, which is also weakly supported. In general, our results show that within the different evolutionary lineages, NET/ATOH8 has experienced a high sequence divergence which makes inferences on basal metazoan relationships difficult, and probably correlates to the notable functional diversification of NET/ATOH8. For instance, NET of fruit fly plays a role in the morphogenesis of wing vein [5], while its vertebrate orthologue ATOH8 is engaged in multiple developmental processes such as neurogenesis [4], [8], development of pancreas [7] and so forth.

It is noteworthy that we did not find any strong support for homologous sequences of NET/ATOH8 in the released genome of *C. elegans* and the other two species of nematodes with tblastn. However, NET of *C. elegans* has been reported previously and referred to as ZK682.4 [2] and T05G5.2 [3] respectively. ZK682.4 has been classified into MyoR family [3] and remains doubtful if it belongs to NET [2], while T05G5.2 is annotated as Achaete-scute transcription factor-related in WormBase (http://www.wormbase.org/; WBGene00001951). Due to such an uncertainty, we excluded the nematodes in our phylogenetic analysis.

### Gene structure of *ATOH8*

With the contribution of our sequencing data, we were able to perform multiple sequence alignments of *ATOH8* at genomic sequence level and protein level. Genomic sequence alignment confirms that vertebrate *ATOH8* consists of three exons, consistent with the annotation from GenBank. Additionally, we found that an alternative third exon may exist in mammals, which has been verified by EST (AL831857.1) from the amygdala of the human brain. Whether this isoform is expressed in other tissues and how it functions is currently unknown. Interestingly, a potential DNA loop may form around exon3b of primate *ATOH8*, which may provide a novel regulation for alternative splicing. Once the loop forms, the access to splicing exon3b may be blocked. As a result, exon3a would have to be utilized during alternative splicing.

The protein alignment shows that in vertebrates the bHLH domain of ATOH8 is highly conserved, suggesting that the bHLH domain of ATOH8 is functionally important in different organisms. However, some variation exists. By sequence comparison, we have found that the frog and zebrafish ATOH8 do not have the proline-rich region, and that only mammals develop the serine-rich region. Such regions could be termed as simple sequence repeats (SSRs). They are evolutionary labile and often variable between species and could fine-tune the function of transcription factors [16]. A few studies have shown that SSRs are functional and could be associated with molecular or morphological divergence: one study shows that stretches of proline and glutamine, when fused to the DNA-binding domain of the GAL4 transcription factor, could activate gene transcription. The activity of transcription increases with repeat length [17]; another study shows that the length of glutamine/alanine-repeats in the gene Runx-2 is correlated with the degree of dorsoventral nose bend and midface length in dogs and other carnivores [18]. It would be very interesting to check whether similar structural differences found in ATOH8 among different species could lead to its functional varieties. We have discovered that the chicken ATOH8 lacks approximately 50 amino acids in the downstream of N-terminus and the serine-rich region compared with mammalian ATOH8. Correspondingly, the functional differences of ATOH8 in the mouse and chicken, according to previous studies are distinguishable. In an earlier study, ATOH8 is shown to promote neurogenesis in the mouse retina [4], while a recent report demonstrates that ATOH8 inhibits neuronal differentiation in the chicken retina [9]. According to the latter study, ATOH8 in the chicken retina functions as a transcriptional activator. In contrast, the mouse ATOH8 acts as a transcriptional repressor in the development of pancreas [7]. Although we cannot exclude other factors such as different cellular contexts coordinating the ATOH8 function, the structural differences of ATOH8 itself in these two species may contribute to the functional divergences. With regard to the extended C-terminus of the predicted opossum ATOH8, direct evidence such as from RT-PCR is required to validate its cDNA sequence.

### Regulatory elements of *ATOH8*

After sequence analysis, we find that the sequences upstream of eutherian mammalian *ATOH8* orthologues are mainly CpG islands without TATA box. The GC content of these islands is more or less commensurate with that of presently reported human and mouse CpG island promoters, approximately 67% and 64% respectively [19]. Instead, the TATA box is present upstream of *ATOH8* orthologues of frog and zebrafish with ordinary GC content, while in chicken, the upstream sequence contains both CpG islands and TATA box. Both CpG islands and TATA box are characteristic of gene promoters [20]. In human genome, about half of the potential promoter regions for human genes are located in CpG islands and about 32% potential promoter regions contain a putative TATA box motif [21]. The transition from a TATA box-type promoter to a CpG island-type promoter reflects the variation of the regulation of *ATOH8* gene expression. TATA box promoters have one single transcription initiation site. Any change in the functional region of the promoter would lead to significant phenotypic alterations. In contrast, CpG island-type promoters usually have multiple transcription start sites (TSS). Which TSS is adopted depends on the cellular status, allowing the fine-tuning of gene expression responding to minor changes in cellular environment [22], [23]. In this way, the CpG island-type promoter of *ATOH8* may be functionally more dynamic than the TATA-box promoter to regulate *ATOH8* expression in a particular cellular environment, thus providing a more specific regulation of *ATOH8* expression. Indeed, the mouse *ATOH8* armed with CpG islands promoter, based on presently available studies, seems more widely expressed during embryonic development than its chicken and zebrafish orthologues. The mouse *ATOH8* transcripts are present in multiple tissues including the developing brain, retina, cortical plates, kidney, pancreas, heart, liver, lung, stomach, intestine and spleen [4], [6], [7]. In comparison, zebrafish *ATOH8* is mainly expressed in the developing retina and skeletal muscle during embryonic development [8]. In chicken, the expression of *ATOH8* is concentrated in the myotome, eyes and podocytes (data not shown). It seems that *ATOH8* expression extends to more organs during evolution. This expansion, if it is true, indicates that *ATOH8* may have multiplied its functions in various organogeneses through evolution, which truly requires the flexibility of the CpG islands promoter to regulate its expression in multiple tissues. For the upstream region of the opossum *ATOH8*, both CpG islands and TATA elements are absent. This may be due to the unique character of the opossum genome. Analysis at genomic level indicates that the opossum has a lower GC content (37.7%) and lower density of CpG islands (0.9%) than other amniotes (40.9%–41.8% GC content and 1.7–2.2% CpG islands density) [24].

Experimentally, we identify that both 944 bp and 442 bp fragments upstream of the start codon of human *ATOH8* are effective in driving the expression of their downstream sequences in the chicken embryo, revealing that the 442 bp fragment at least contains effective if not optimal components as the promoter of *hATOH8*. It also tells us that the CpG islands type promoter of human *ATOH8* can function in the chicken cells. It will be desirable to know if the TATA promoter of *ATOH8* can work in the mammals as well.

### Remote regulation of gene expression in human chromosome 2

Comparative genomics have shown that human and chimpanzee genome sequences are nearly identical with only 1.23% nucleotide divergences [25]. The focus of study thus turns to differences rather than similarities of the genome between human and chimpanzee. The most striking difference is the reduction of the chromosome from n = 24 in the chimpanzee to n = 23 in the human, caused by the fusion of two medium-sized acrocentric chromosomes in the chimpanzee that give rise to the long and short arms of the large submetacentric human chromosome 2, the human “fusion chromosome 2” [26], [27], [28], [29]. This difference between human and chimpanzee at chromosome level may be one of the reasons causing the divergence of the two species.

In the interphase nucleus, chromosomes with corresponding proteins occupy discrete territories. Recently, interchromosomal and intrachromosomal interactions during interphase have been shown to regulate gene transcription in the mammalian nucleus [30]. To quote a few, the expression of olfactory receptor genes is regulated by the association of their promoters with loci from another chromosome [31]; interaction of different loci within one single chromosome is implicated in the DNA recombination to attain antigen specificity of B and T cells [32], [33]. It is proposed that the distant interchromosal and intrachromosomal interactions render remote regulatory elements and corresponding proteins to approach closely their target genes, thus coordinating the expression of these genes [34]. From our chromosome painting data, we demonstrated that human chromosome 2 containing the *ATOH8* gene in its short arm is located in a restricted territory in the nuclei of interphase cells, while in chimpanzee, orangutan, rhesus monkey and marmoset the two chromosomes which are respectively orthologous to the long and short arms of human fusion chromosome 2 are within separate chromatin territories in interphase nuclei. Based on the hypothesis mentioned above, the differences of gene components in the chromosome(s) and protein components in corresponding chromosome territory(ies) between human and non-human primates, resulting from the fusion of two chromosomes, may differentially regulate the expression of genes, including *ATOH8*, in the relevant chromosome(s).

In summary, we analyzed the phylogeny of *ATOH8* gene, and revealed the diversification of this gene across different species, especially for the vertebrate orthologues. Such diversification may significantly and delicately contribute to the various functions of *ATOH8* in different cellular contexts.

## Methods

### Resequencing of *ATOH8* gene loci and cDNA of several species

Based on known sequence information of *ATOH8* gene loci of several species, we designed primers to perform genomic PCR to fill sequence gaps of *ATOH8* gene loci for the chimpanzee, rhesus monkey, dolphin, horse, dog and cat. Genomic DNA was extracted from blood with Quick-gDNA mini prep kit (Zymo Research, Germany). PCRs were performed with Hot-start PCR kit (Qiagen). PCR products were directly sequenced or cloned into pDrive vector (Qiagen) for subsequent sequencing. ‘Primer walking’ strategy was adopted to sequence through the whole length of the PCR products. To complete missing sequence of the chicken *ATOH8* gene locus, Southern blot was conducted following previously described protocols [35] from chicken BAC clone 13M24 (provided by Prof. Dr. MA Grownen, Wageningen Agricultural University, the Netherlands). The BAC DNA was digested with *KpnI* or/and *EcoRI* and separated on 0.8% agarose gel. The digoxigenin (DIG) labelled probe was synthesized with PCR using the conserved exon 1 of human or frog *ATOH8* as the template. Based on results from Southern blot, a 3.4kb fragment was selected and cloned into pDrive vector (Qiagen) for sequencing, which proved to contain the upstream region and the first exon of chicken *ATOH8*. For RT-PCR, the chicken and frog total mRNA was extracted from chicken and frog embryos with Trizol (Sigma). Primers were designed and RT-PCR was performed with one-step RT-PCR kit (Qiagen).

### Bioinformatic sequence analysis

Searches for sequences homologous to NET/ATOH8 in invertebrates were performed in GenBank using tBLASTn (http://www.ncbi.nlm.nih.gov/blast/Blast.cgi?). Multiple gene loci sequence comparisons of *ATOH8* from different species were performed with the mVista program provided by VISTA (http://genome.lbl.gov/vista/index.shtml). Length of CpG islands of upstream region of *ATOH8* was evaluated by CpG islands searcher (http://cpgislands.usc.edu/).

### Construction of *ATOH8* gene tree

In this study, two phylogenetic data sets have been analyzed. First, an *ATOH8* cDNA sequence alignment of 18 vertebrate species along with the *ATOH8* orthologues from *Ciona intestinalis* and *Drosphila melanogaster* was carried out using the translation alignment method and the software MAFFT version 6 program [36] (E-INS-I and L-INSI-algorithms) implemented as plugin in the Geneious software [37]. Second, a protein sequence alignment was constructed of species from several evolutionarily very different lineages bearing the ATOH8/NET-like bHLH motif from examined metazoan species and the ancestral protein Amq1 from sponge as outgroup with MAFFT. Reliably aligned blocks of the very divergent protein sequences were extracted from the alignment using the software gBlocks version 0.91 [38].

An appropriate nucleic and amino acid substitution model was determined using jModeltest version 0.1.1 [39], [40] and ProtTest version 2.4 [41]. MrBayes version 3.1.2 [42], RAxML version 7.2.7 [43] and PhyML [44] for the protein data set were employed to estimate phylogenetic trees. Bayesian analyses with MrBayes were conducted by computing 10×10^6^ Markov Chain Monte Carlo generations in four parallel runs, each with four chains. Trees were sampled every 100^th^ generation. Convergence was determined by comparing the split frequencies of the runs and the first 1000 trees were excluded for the nucleotide and protein alignment. Using RAxML, the most likely tree was determined by conducting 10 slow ML searches on 10 randomized stepwise addition parsimony trees. Furthermore, a total of 10000 rapid bootstrap replicates have been computed with RAxML. These were mapped onto the MrBayes tree. For PhyML, branch length and topology optimization were used and 1000 bootstrap replicates were performed. For both alignments the Bayesian trees were shown in this study and branches with posterior probabilities below 0.75 were collapsed to polytomies to display only trees with strong phylogenetic signal.

### Construction of *ATOH8* promoter- EGFP reporter system

Human BAC clone (RZPDB737E072124D) containing *hATOH8* gene locus was purchased from Germany Genome Resource Center and cultured in 200ml LB-medium with 100 µg/ml chloramphenicol at 30°C for 48 hours. The plasmid DNA was isolated using Large Construct Kit (Qiagen) in accordance with the manufacturer's instructions. The DNA was digested with *KpnI* and separated on 0.8% agarose gel. A band (8.5 kb) was isolated from the gel and cloned into *KpnI* pre-linearised pDrive vector. The ends of the expected insert in the plasmid have been sequenced for confirmation of the presence of human *ATOH8* gene locus. The cloned fragment (8.533 kb) covers the region from 8.3 kb upstream to 217 bp downstream of the *hATOH8* start codon. A 944 bp and a 442 bp fragment upstream of *hATOH8* start codon derived from the 8.5 kb fragment were pieced together with *hATOH8* coding sequence, and cloned into pIRES2-EGFP (Clontech) vector by replacing the CMV promoter respectively. The control plasmid was constructed by replacement of the CMV promoter with an adaptor (adaptor sequence: Forward: 5′ CTAGCTAAGC TTGCCCGCGC CATGAAGCAC ATCCCGGTCC 3′; and Reverse: 5′ TCGAGGACCG GGATGTGCTT CATGGCGCGG GCAAGCTTAG 3′). The constructed plasmids were sequenced for verification (Eurofins, Germany).

### *In ovo* electroporation and imaging of embryos

Fertilized chicken eggs were incubated to HH stage 17–18. The embryo was exposed by opening a window in the egg shell. 3–6µg/µl of DNA plasmids was electroporated into somites of the chicken embryo according to procedures described [45]. After 12–16 hours of re-incubation, the embryos were photographed under an epifluorescence microscope (MZFLIII, Leica) attached with a digital camera (DC300F, Leica).

### *In situ* hybridization

A 1.3 kb human *ATOH8* cDNA fragment cloned into pDrive was used as template to synthesize digoxigenin-labelled RNA probe. The plasmid was linearised with *XbaI* and subsequent in vitro transcription was performed with T7 RNA polymerase for the synthesis of antisense probe. *BamHI* was used to linearize the plasmid to synthesize sense probe as control with SP6 RNA polymerase. Fertilized chicken eggs obtained from a local breeder were incubated at 38°C, 80% humidity. Embryos were staged as described [46], sacrificed and fixed in 4% PFA/PBT. Whole mount *in situ* hybridization was performed as described previously [47].

### FISH and chromosome painting

To perform the comparative FISH on *ATOH8* gene location in chromosomes of human and non-human primates, the FISH probes were prepared from the human BAC clone (RP11-439L14) containing whole *hATOH8* gene locus. The human BAC clone DNA which contains *hATOH8* gene was isolated using a Large Construct Kit (Qiagen). The probe labelling was performed via PCR with random primers. The FISH processes was according to the protocols as described previously [48]. Briefly, the peripheral lymphocytes from human *(Homo sapiens, HSA)*, chimpanzee *(Pan troglodytes, PTR)*, orangutan *(Pongo pygmaeus, PPY)*, rhesus macaque *(Macaca mulatta, MMU)* and lymphoblastoid cells from the common marmoset *(Callithrix jacchus, CJA)* were cultured. The preparation of chromosomes of human and non-human primates was carried out on the pre-treated glass slides. The human whole-chromosome painting probe for human chromosome 2 [48] was used to unequivocally assign hybridization signals to human chromosome 2 and the orthologous chromosomes in non-human primates, either for the territories in interphase cells or for the packaged chromosomes in metaphase cells. The new orthologous numbering is used for chimpanzee chromosomes in accordance to McConkey's suggestion [48]. The slides were counterstained with DAPI (4′, 6-diamidino-2-phenylindole; 0.14 µg/ml) and mounted in Vectashield (Vector Laboratories). Results were evaluated using a Zeiss Axiophot epifluorescence microscope equipped with single-bandpass filters for excitation of red, green, and blue (Chroma Technologies, Brattleboro, VT). During exposures, only excitation filters were changed allowing for pixel-shift-free image recording. Images of high magnification and resolution were obtained using a black-and-white CCD camera (Photometrics Kodak KAF 1400; Kodak, Tucson, AZ) connected to the Axiophot.

## Supporting Information

Figure S1 **Ensembl species tree.** The species tree represents the mostly accepted phylogeny of analyzed species and is provided by Ensembl. Species used for analysis of *ATOH8* phylogeny is marked with red.
(TIF)

Figure S2 **The adapted phylogenetic tree of** ***ATOH8*** **gene constructed by Ensembl.** The tree was generated using TreeBeST pipeline. The tree is based on an *ATOH8* sequence alignment of *ATOH8* orthologues detected in the Ensembl genome database.
(TIF)

Figure S3 **The distribution of CpG islands in the upstream region of** ***ATOH8.*** The length of CpG islands in the *ATOH8* upstream region of 17 vertebrate species is presented. In opossum, frog and zebrafish, there are no CpG islands in the upstream region of *ATOH8.*
(TIF)

Table S1 **The location of loop donor (LD) in chromosomes of primates.**
(DOC)

Table S2 **Accession numbers of ATOH8/NET cDNA and protein orthologues.**
(DOC)

Table S3 **Sequences of NET orthologues used for phylogenetic analysis.**
(XLS)
