# Supplementary figures and images for: Diversification and Molecular Evolution of ATOH8, a Gene Encoding a bHLH Transcription Factor
Source: PLoS One. 2011 Aug 4;6(8):e23005. doi: 10.1371/journal.pone.0023005 (PMC3150394; doi:10.1371/journal.pone.0023005)

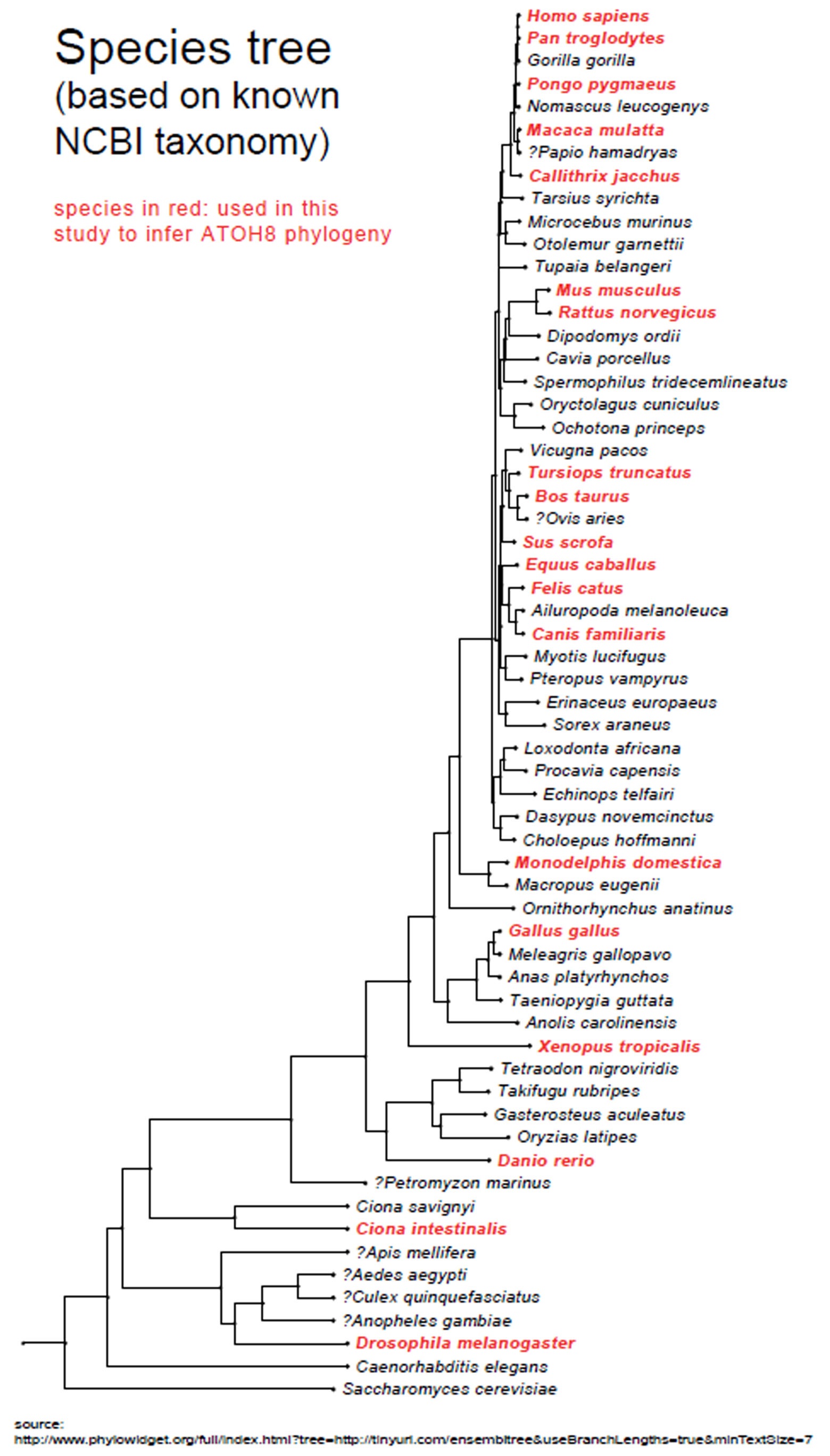

Supplement: Figure S1 — Ensembl species tree. The species tree represents the mostly accepted phylogeny of analyzed species and is provided by Ensembl. Species used for analysis of ATOH8 phylogeny is marked with red. (TIF) [file pone.0023005.s001.tif]

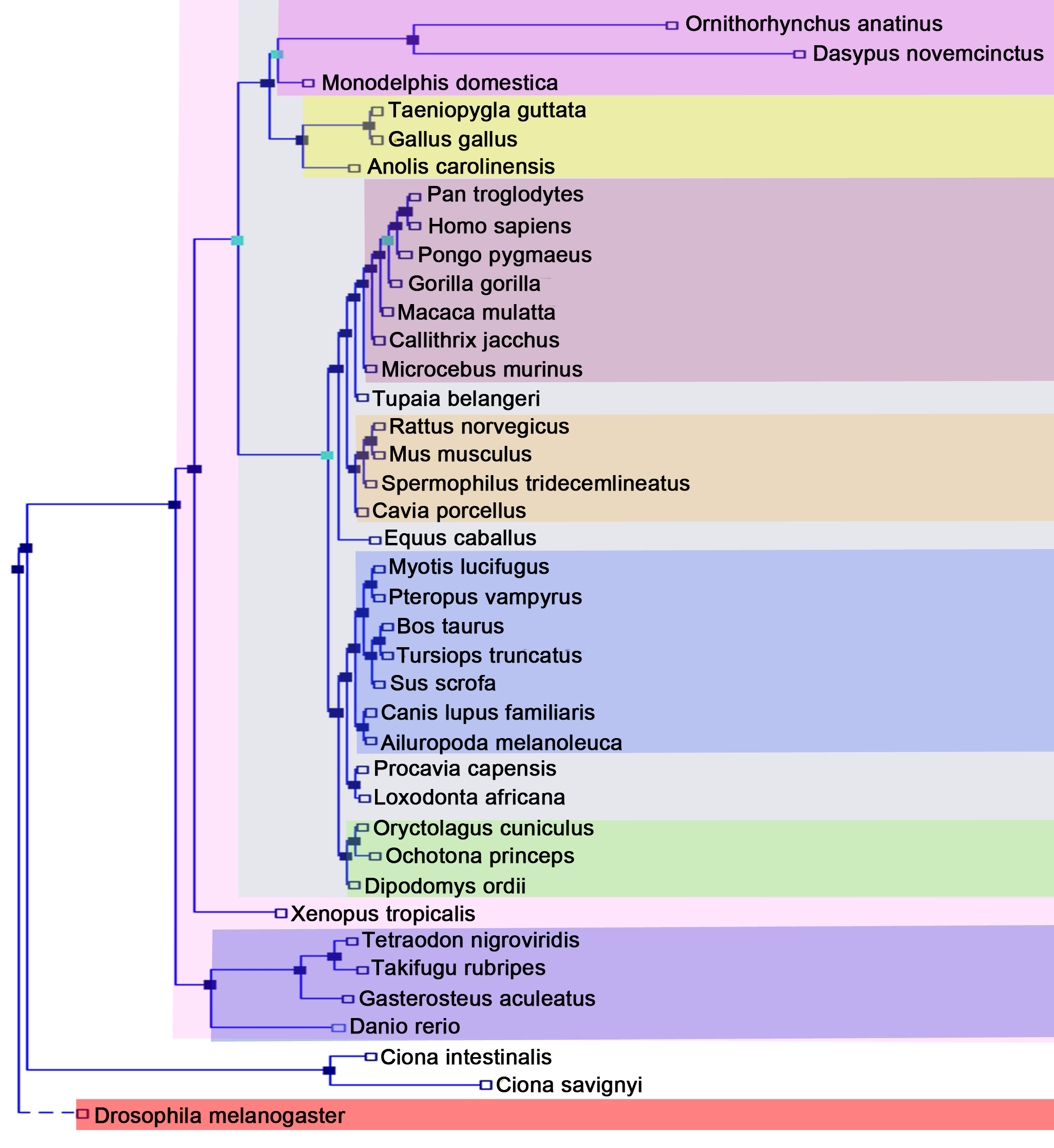

Supplement: Figure S2 — The adapted phylogenetic tree of ATOH8 gene constructed by Ensembl. The tree was generated using TreeBeST pipeline. The tree is based on an ATOH8 sequence alignment of ATOH8 orthologues detected in the Ensembl genome database. (TIF) [file pone.0023005.s002.tif]

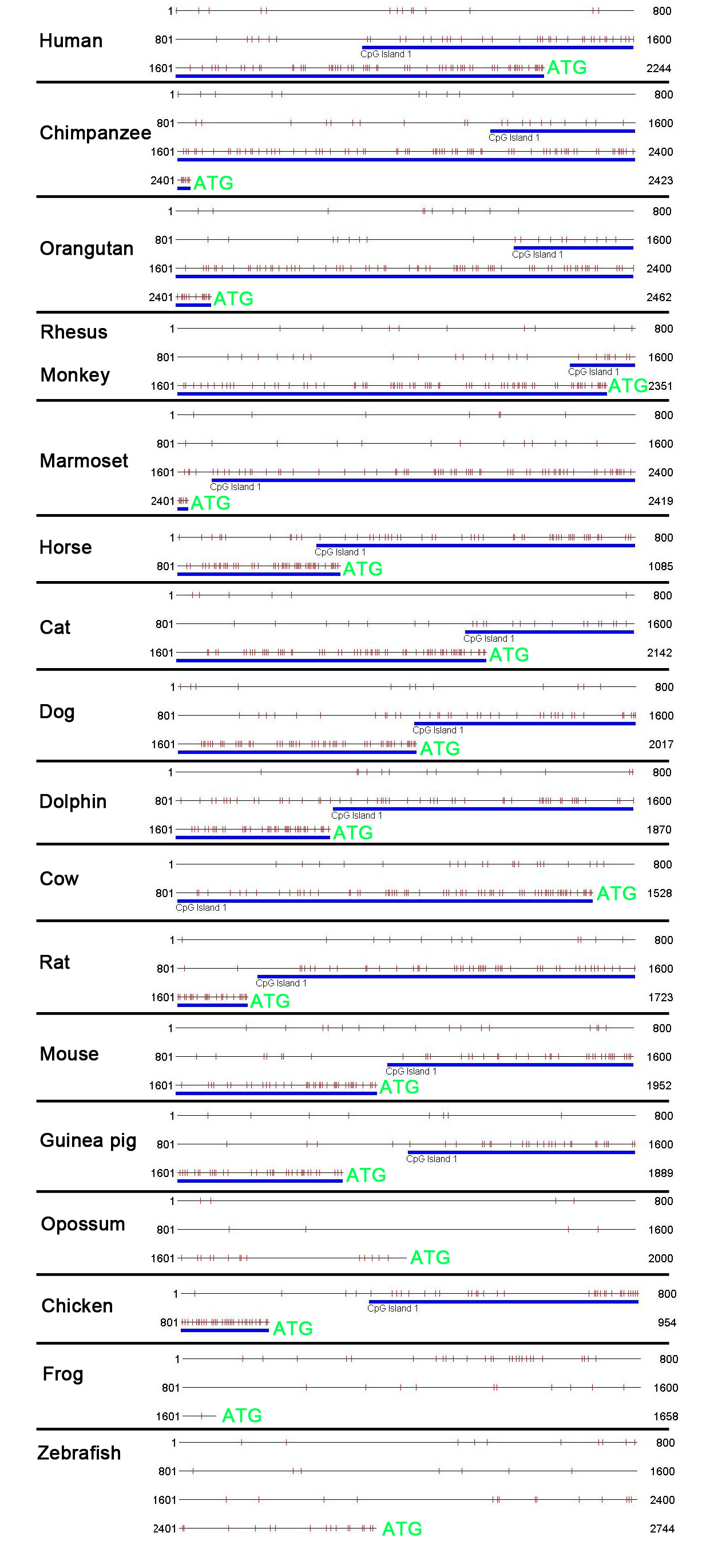

Supplement: Figure S3 — The distribution of CpG islands in the upstream region of ATOH8. The length of CpG islands in the ATOH8 upstream region of 17 vertebrate species is presented. In opossum, frog and zebrafish, there are no CpG islands in the upstream region of ATOH8. (TIF) [file pone.0023005.s003.tif]
