# Supplementary material for: Diversification and Molecular Evolution of ATOH8, a Gene Encoding a bHLH Transcription Factor
Source: PLoS One. 2011 Aug 4;6(8):e23005. doi: 10.1371/journal.pone.0023005 (PMC3150394; doi:10.1371/journal.pone.0023005)
Supplement: Table S1 — The location of loop donor (LD) in chromosomes of primates. (DOC) [file pone.0023005.s004.doc]

Supplementary Table 1. The location of loop donor (LD) in chromosomes of primates.

|  | **Location** | | **Blast matching** | | |
| --- | --- | --- | --- | --- | --- |
| ***Taxo*** | **Start** | **End** | **Length** | **Ratio** | **Score** |
| ***Homo sapiens*** | Chr2: 85865749 | Chr2: 85865863 | 115 | 77.0% | 96 |
| ***Pan troglodytes*** | Chr2A: 87704317 | Chr2A: 87704421 | 105 | 88.6% | 89 |
| ***Pongo pygmaeus abelii*** | Chr2A: 24367225 | Chr2A: 24367329 | 105 | 87.8% | 81 |
| ***Callithrix jacchus*** | Contig 669: 174660 | Contig 669: 174756 | 97 | 88.6% | 83 |
| ***Macaca mulatta*** | Chr13:96363376 | Chr13:96363441 | 66 | 87.9% | 58 |
