# Supplementary material for: Diversification and Molecular Evolution of ATOH8, a Gene Encoding a bHLH Transcription Factor
Source: PLoS One. 2011 Aug 4;6(8):e23005. doi: 10.1371/journal.pone.0023005 (PMC3150394; doi:10.1371/journal.pone.0023005)
Supplement: Table S2 — Accession numbers of ATOH8/NET cDNA and protein orthologues. (DOC) [file pone.0023005.s005.doc]

**Supplementary Table 2 Accession numbers of ATOH8/NET cDNA and protein orthologues**

| **Common name** | **Species** | **Accession number** | |
| --- | --- | --- | --- |
|  |  | **mRNA** | **Protein** |
| Human | *Homo sapiens* | NM_032827.6 | [NP_116216.2](http://www.ncbi.nlm.nih.gov/protein/NP_116216.2) |
| Chimpanzee | *Pan troglodytes* | BN001499 | CBN67691.1 |
| Orangutan | *Pongo abelii* | BN001494 | CBM40483.1 |
| Rhesus monkey | *Macaca mulatta* | BN001501 | CBN67694.1 |
| Marmoset | *Callithrix jacchus* | BN001495 | CBM40484.1 |
| Cat | *Felis catus* | BN001498 | CBN67684.1 |
| Dog | *Canis lupus familiaris* | BN001500 | CBN67693.1 |
| Cattle | *Bos taurus* | [XM_868199.4](http://www.ncbi.nlm.nih.gov/nuccore/XM_868199.4) | XP_873292 |
| Pig | *Sus scrofa* | BN001502 | CBN67697.1 |
| Dolphin | *Tursiops truncatus* | BN001503 | CBN67702.1 |
| Horse | *Equus caballus* | BN001504 | CBN67705.1 |
| Guinea pig | *Cavia porcellus* | BN001493 | CBM40482.1 |
| Rat | *Rattus norvegicus* | [NM_001109241.1](http://www.ncbi.nlm.nih.gov/nuccore/NM_001109241.1) | NP_001102711 |
| Mouse | [*Mus musculus*](http://www.ncbi.nlm.nih.gov/Taxonomy/Browser/wwwtax.cgi?mode=Undef&name=Mus+musculus&lvl=0&srchmode=1&keep=1&unlock) | [NM_153778.3](http://www.ncbi.nlm.nih.gov/nuccore/NM_153778.3) | NP_722473 |
| Opossum | *Monodelphis domestica* | XM_001376335.1 | XP_001376372.1 |
| Chicken | *Gallus gallus* | FN868883 | CBM40860.1 |
| Frog | [*Xenopus tropicalis*](http://www.ncbi.nlm.nih.gov/Taxonomy/Browser/wwwtax.cgi?mode=Undef&name=Xenopus+laevis&lvl=0&srchmode=1&keep=1&unlock) | FN868884 | CBM40861.1 |
| Zebrafish | *Danio rerio* | [NM_001079991.2](http://www.ncbi.nlm.nih.gov/nuccore/NM_001079991.2) | NP_001073460 |
| Sea squirt | *Ciona intestinalis* | [XM_002122470.1](http://www.ncbi.nlm.nih.gov/nuccore/198419573) | [XP_002122506.1](http://www.ncbi.nlm.nih.gov/protein/198419574) |
| Fruit fly | *Drosophila melanogaster* | [NM_080081.2](http://www.ncbi.nlm.nih.gov/nuccore/NM_080081.2) | [NP_524820.2](http://www.ncbi.nlm.nih.gov/protein/NP_524820.2) |
